# Parental personality disorder symptoms and children’s social skills: a prospective community study

**DOI:** 10.1007/s00787-022-01965-0

**Published:** 2022-03-02

**Authors:** Lars Wichstrøm, Anna Emilie Borgen, Silje Steinsbekk

**Affiliations:** 1 Department of Psychology, Norwegian University of Science and Technology, Trondheim, Norway; 2 Department of Child and Adolescent Psychiatry, St. Olavs University Hospital, Trondheim, Norway

**Keywords:** Middle childhood, Longitudinal, Personality disorder, Prospective, Social skills

## Abstract

Acquiring age-appropriate social skills, arguably a major prerequisite for favorable psychosocial development in children, is targeted in a range of interventions. Hence, identifying factors that limit this acquisition may inform preventative and treatment efforts. Personality disorders are characterized by pervasive and enduring dysfunctional interpersonal functioning, including parenting, and could thus entail risk for offspring in not developing adaptive interpersonal skills. However, no study has tested this possibility. A representative sample drawn from two birth cohorts of Norwegian 4-year-olds (*n* = 956) and their parents was followed up at ages 6, 8, and 10 years. Parents’ personality disorder symptoms were measured dimensionally with the DSM-IV and ICD-10 Personality Questionnaire, and children’s social skills were evaluated by the Social Skills Rating System. A difference-in-difference approach was applied to adjust for all unmeasured time-invariant confounders, and parental symptoms of depression and anxiety were entered as covariates. Increased Cluster B symptoms in parents of children aged 4 to 6 years predicted decreased social skill development in offspring (*B* = −0.97, 95% CI −1.58, −0.37, *p* = 0.002). On a more granular level, increased symptoms of borderline (*B* = −0.39, CI −0.65, −0.12, *p* = 0.004), histrionic (*B* = −0.55, CI −0.99, −0.11, *p* = 0.018), and avoidant (*B* = −0.46, CI−0.79, −0.13, *p* = 0.006) personality disorders in parents predicted decreased social skill development in offspring. Subclinical levels of borderline, histrionic and avoidant personality disorders in parents may impair the development of social skills in offspring. Successfully treating these personality problems or considering them when providing services to children may facilitate children’s acquisition of social skills.

## Introduction

Social competence is the ability to interact successfully and effectively with others [37], and social skills contribute to this efficacy. Attaining social skills is a significant developmental task and is considered an essential component of healthy functioning, development and adjustment, as indicated by its ability to predict high academic achievement [9], better friendships and peer acceptance [44], and favorable mental health [7]. Moreover, improving social skills is a prime focus for a range of interventions for a multitude of psychopathologies. Therefore, to aid the development of effective health and educational policies, as well as preventative and treatment efforts, identifying determinants of social skills is a vital endeavor. Parental mental health problems have been identified as one factor limiting the acquisition of social skills in offspring [11]. To date, attention has predominantly been drawn to parental depression [24, 50] and anxiety [13]. By focusing extensively on emotional and behavioral disorders, researchers may have overlooked the potential contribution from other conditions.

Social skills are learned, shaped, and influenced through interaction with competent partners, such as parents [31]. Parents are believed to help children learn social skills through their sensitive use of practices, such as induction, feedback, social coaching, and monitoring of their child’s interactions with others [31, 39]. In contrast, insensitive, unresponsive, inconsistent, harsh, or punitive parents may not only hinder the learning of prosocial behavior [36] but also directly model socially incompetent or even antisocial behavior [8]. Personality disorders (PDs) are characterized by enduring dysfunctional interpersonal patterns, deviant cognitive and affective modulation, low impulse control, unstable emotion regulation and affective lability [3]. Hence, it seems reasonable to hypothesize that parental PDs interfere with parenting in a way that is decisive for offspring social skills acquisition. This contention was supported by multiple reviews and a meta-analysis concluding that paranoid, schizotypal, borderline, and avoidant PDs are indeed associated with impairments in parenting and parent–child interactions [40, 49], whereas the other PDs were not associated with impairments in the parent–child domain. Notably, because research on PDs other than borderline and antisocial PDs (both belonging to Cluster B) is limited, any null effects in such summaries of previous findings should be interpreted cautiously; thus, available findings do not allow for specific hypothesis about differential effects of different PDs on offspring social skills. No study has investigated whether symptoms of parental PDs might limit the development of social skills in children, a task that we undertake herein.

The above indirect evidence linking parental PDs to offspring social skills stems from observational research. Both adult PDs [23] and child social skills [20] are heritable. Hence, although not directly examined, these heritabilities may overlap, and the associations between parental PDs and child social skills may be due to passive gene-environment correlation. To adjust for the impact of time-invariant between-person confounding (e.g., genes), we therefore test whether changes in parental PD symptoms predict changes in children’s social skills [1]. Even so, the findings may still be influenced by time-varying confounders. We therefore correct for the arguably most likely time-varying confounders [17], namely parental depression and anxiety. While the DSM-5 conceptualizes PDs as categorical, evidence strongly suggests that they are continuous in nature [47]. Consequently, we examine PDs dimensionally. Moreover, describing the 10 PDs as belonging to three clusters (A = odd-eccentric: paranoid, schizoid, and schizotypal; B = dramatic, emotional, or erratic: antisocial, borderline, histrionic, and narcissistic; C = anxious or fearful: avoidant, dependent, and obsessive–compulsive) may prove useful in the current context, as the PDs within each cluster share some of the same interpersonal features and may hence affect children’s social skills in similar ways. Symptoms of PDs are therefore examined both cluster-wise and individually. Given the above preliminary evidence, we hypothesize that an increase in symptoms of parental PDs when the child is aged 4–6 years will predict a decline in social skills in the child from age 4–10 years.

## Methods

### Procedure and participants

The 2003 and 2004 birth cohorts living in Trondheim (*N* = 3456), Norway, were asked to participate in the Trondheim Early Secure Study (TESS) [42] through a letter of invitation that included the Strength and Difficulties Questionnaire for 4- to 16-year-olds, a screening tool for mental health problems (SDQ) [19, 43]. Parents were asked to complete the questionnaire and bring it with them when attending the well-child clinic for a regular health check for children at age 4 years (3358 attended). Parents who lacked sufficient proficiency in Norwegian to fill out the SDQ were excluded (*n* = 176), and 166 parents were missed being asked. Health nurses informed the parents about the study and obtained their written consent to participate (*n* = 2477; 82.2% consent rate). To increase variability and statistical power, children with behavioral and emotional problems were oversampled by allocating children to four strata based on their SDQ scores (cut-offs: 0–4, 5–8, 9–11 and 12–40). This oversampling was accounted for in the analyses. The likelihood of being included in the study increased as SDQ scores increased (0.37, 0.48, 0.70, and 0.89 in the respective strata). Thus, 1,250 participants were randomly drawn, and 1,007 were successfully examined in the first wave (T1; *M*_age_ = 4.59, SD = 0.25). Children were followed up at ages 6 (T2; *n* = 795; *M*_age_ = 6.72, *SD* = 0.19), 8 (T3; *n* = 699; *M*_age_ = 8.79, SD = 0.23) and 10 (T4; *n* = 702; *M*_age_ = 10.51, *SD* = 0.17). Families with valid data from at least one wave were included and formed the analytical sample (*n* = 956; 49.1% boys). Parental PDs were examined at T1 and T2. Little’s MCAR test [28] including all study variables strongly suggested that data were missing completely at random, *χ*^2^ = 1785.23, *df* = 7,064,* p* = 1.00. In 44 cases, the parent accompanying the child at T2 was not the same as the parent at T1, and in those cases, values were considered missing at T2. The sample, adjusted for stratification, was similar to the population of parents of 4-year-olds in Trondheim with respect to educational level, employment and type of position, but contained more divorced parents (6.8%) than the population (2.1%). The population of Trondheim shares several similarities with the average Norwegian population in terms of employment (equal to the national rate), average gross income (99.5% of the national average) and households (two-parent families account for 80.0% of the households compared to a national average of 81.4%) [46].

### Measures

Parental symptoms of personality disorders: The DSM-IV and ICD-10 Personality Questionnaire (DIP-Q) [33] is a self-report questionnaire consisting of 140 items scored categorically (true/false) to capture both categorical diagnoses and dimensional measures of all ten DSM-IV and all eight ICD-10 personality disorders. The number of scores was summed. For diagnostic purposes, a five-item distress/impairment scale is also included along with the Global Assessment of Functioning (GAF) scale [2]. A score of ≥ 2 on the distress/impairment scale and a GAF score of ≤ 70 is required for a diagnosis to be set. Favorable reliability and validity in clinical as well as non-clinical samples have been documented [33–35]. Because item responses were dichotomous, an ordinal theta coefficient was used to examine reliability [51]. The levels of internal consistency for Clusters A, B and C at the two time points were *θ* = 0.89/0.88; *θ* = 0.95/0.95; and *θ* = 0.89/0.88. With respect to individual PDs, acceptable values were identified as follows: paranoid: *θ* = 0.88/0.82; schizoid: *θ* = 0.70/0.70; schizotypal: *θ* = 0.90/0.87; antisocial: *θ* = 85/0.80; borderline: *θ* = 0.91/0.90; histrionic: *θ* = 0.76/0.78; narcissistic: *θ* = 0.77/0.77; avoidant: *θ* = 0.87/0.89; dependent: *θ* = 0.75/0.74; and obsessive–compulsive: *θ* = 0.71/0.71.

Social skills in children: Parents evaluated their children’s social skills using the Social Skills Rating System (SSRS) [21]. The SSRS measures four major social skills with 10 items each on a four-point scale that form the basis of socially competent behavior that facilitates social interactions in different social settings: cooperation, assertiveness, self-control and responsibility. The reliability and validity of SSRS have been consistently established through a wide range of studied across the globe (e.g., [22]). Given that we had no specific hypotheses for the separate dimensions, the SSRS total scale was applied (*α* = 0.89–0.92).

Parental symptoms of depression and anxiety: The Beck Depression Inventory-II [5], *α* = 0.87/0.87, and the Beck Anxiety Inventory [4], *α* = 0.82/0.87 are 21-item measures applying a 4-point scale and were used to measure the covariates parental symptoms of depression and anxiety. Their psychometric properties have been extensively documented in a range of studies [5, 18].

### Statistical analysis

Analyses were conducted using Mplus version 8.1 [32] Population weights were applied to arrive at correct population estimates, corresponding to the number of children in the population in a stratum divided by the number of participants in that stratum. A robust maximum likelihood estimator (MLR) that does not presuppose multivariate normality was used. The estimator also provides robust standard errors. Missing-ness was handled according to a full information maximum likelihood procedure using all available data.

**Fig. 1:**
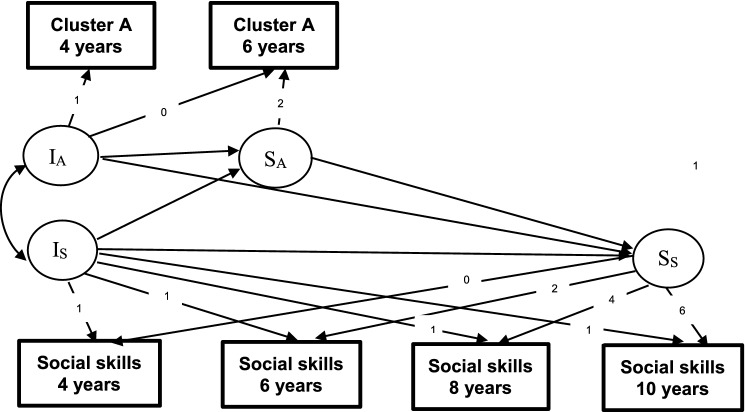
Conceptual model of relation between change and level in parental symptoms of personality disorder clusters and offspring social skills. Co-varying parental anxiety and depression as well as error terms are not depicted. Personality disorder clusters are represented with cluster A. *I*_*A*_ intercept for cluster A, *S*_*A*_ slope for cluster A, *I*_*S*_ intercept for social skills, *S*_*S*_ slope for social skills

To adjust for time-invariant confounding, we applied a change–predicting–change approach, which is essentially a fixed-effects analysis [1], using latent growth curves. In this model, each child’s development over time is modeled by two latent constructs, the intercept (level, relative to others) and slope (change, relative to oneself). Intercepts were set at T1, and the slopes were represented as yearly changes. To accommodate growth curve analysis with two measurement points (i.e., parental PD symptoms, depression, and anxiety), the variances in observed PDs at each point were set to zero, thereby transferring the variance to the latent constructs of interest (intercept and slope). We first tested a model comprising the three clusters, anxiety and depression. All slopes were regressed on all intercepts to control for regression toward the mean effects. Changes (latent growth) in social skills were regressed on the number of symptoms (intercept) and changes in the number of symptoms (slope) in each cluster, anxiety, and depression. All slopes and all intercepts were allowed to correlate, see Fig. 1 for a simplified version of the model. Second, we examined the effect of symptoms of specific PDs in three separate models—one for each cluster.

## Results

At T1 and T2, respectively, 8.8% (95% CI: 7.7–10.2 and 10.4% (95% CI: 9.1–12.0) fulfilled the criteria for at least one personality disorder. The level of social skills increased from age 4 to 10 years (Table 1), as reflected in an unconditional latent growth curve model (yearly increase = 4.80, *p* < 0.001). With respect to parental PD clusters, the mean number of symptoms was highest for parental PD Cluster C at both the initial testing and 2 years later. Except for a negligible decrease in Cluster B symptoms (yearly change = −0.02, *p* = 0.034), the number of symptoms did not change from T1 to T2 at the group level. Even so, variances in intra-individual changes were substantial (social skills = 1.15, *p* = 0.016, Cluster A = 0.17, *p* < 0.001, Cluster B = 0.09, *p* < 0.001, Cluster C = 0.17, *p* < 0.001).

**Table 1:** Means and standard deviations for the study variables

Variable	Mean	Standard deviation
Social skills—age 4	50.08	8.29
Social skills—age 6	73.61	13.10
Social skills—age 8	74.37	13.35
Social skills—age 10	78.06	13.45
Cluster A—age 4	0.92	1.03
Cluster A—age 6	0.89	1.03
Cluster B—age 4	1.25	0.70
Cluster B—age 6	1.20	0.69
Cluster C—age 4	1.48	0.97
Cluster C—age 6	1.48	0.98
Parental anxiety—age 4	1.81	2.95
Parental anxiety—age 6	2.25	3.59
Parental depression—age 4	4.03	4.90
Parental depression—age 6	4.61	5.53
Paranoid—age 4	0.85	1.20
Paranoid—age 6	0.83	1.81
Schizoid—age 4	0.54	0.79
Schizoid—age 6	0.55	0.84
Schizotypal—age 4	1.37	1.72
Schizotypal—age 6	1.29	1.66
Antisocial—age 4	0.64	0.78
Antisocial—age 6	0.63	0.82
Borderline—age 4	1.18	1.62
Borderline—age 6	1.13	1.54
Histrionic—age 4	1.45	0.78
Histrionic—age 6	1.40	0.76
Narcissistic—age 4	1.73	0.98
Narcissistic—age 6	1.67	0.96
Avoidant—age 4	0.76	1.26
Avoidant—age 6	0.78	1.28
Dependent—age 4	0.85	1.06
Dependent—age 6	0.84	1.02
Obsessive–compulsive—age 4	2.83	1.63
Obsessive–compulsive—age 6	2.80	1.65

Clusters of PDs: A change–predicting–change model including the three clusters did fit the data well, *χ*^2^ = 72.63, *df* = 28, *p* < 0.001, CFI = 0.986, TLI = 0.954, RMSEA = 0.041 (90% CI 0.029, 0.052). Increased parental symptoms of Cluster B PDs, but not Cluster A or C PDs, predicted decreased social skills when adjusted for the social skills intercept (level) and intercept and slope (change) in parental depression and anxiety (Table 2). Higher levels of social skills were correlated with fewer symptoms of Cluster A (*r* = −0.08, *p* = 0.013), B (*r* = −0.09, *p* = 0.015), and C (*r* = −0.16, *p* < 0.001) PDs, as well as less anxiety (*r* = −0.11, *p* < 0.001) and depression (*r* = −0.11, *p* = 0.001) in parents.

**Table 2:** Change in social skills from age 4 to 10 years predicted from level (age 4) and change from age 4 to 6 in parental personality disorder symptom clusters, anxiety and depression

Predictor	*B* (95% CI)	*β*	*p*-value
Intercept social skills	0.14 (−0.03, 0.31)	−0.12	0.11
Cluster A—intercept	−0.11 (−0.33, 0.11)	−0.11	0.32
Cluster A—slope	0.02 (−0.47, 0.51)	0.01	0.94
Cluster B—intercept	−0.28 (−0.62, 0.05)	−0.18	0.093
Cluster B—slope	−0.97 (−1.58, −0.37)	−0.26	0.002
Cluster C—intercept	0.02 (−0.29, 0.34)	0.02	0.89
Cluster C—slope	−0.33 (−0.79, 0.14)	−0.12	0.17
Parental anxiety—intercept	−0.02 (−0.10, 0.07)	−0.05	0.88
Parental anxiety—slope	−0.05 (−0.16, 0.06)	−0.09	0.34
Parental depression—intercept	0.02 (−0.03, 0.07)	0.08	0.71
Parental depression—slope	0.02 (−0.06, 0.09)	0.04	0.65

Individual PDs: Low levels (intercept) of social skills correlated with more symptoms (intercepts) of schizoid (*r* = −0.11, *p* = 0.008), borderline (*r* = −0.12, *p* = 0.001), avoidant (*r* = −0.15, *p* < 0.001), dependent (*r* = −0.18, *p* < 0.001), and obsessive–compulsive (*r* = −0.09, *p* = 0.024) PDs—but not with symptoms of schizotypal (*r* = −0.06, *p* = 0.095), paranoid (*r* = −0.06, *p* = 0.095), antisocial (*r* = −0.01, *p* = 0.93), histrionic (*r* = −0.02, *p* = 0.64), and narcissistic (*r* = −0.04, *p* = 0.31) PDs.

With respect to symptoms of individual PDs predicting changes in social skills, increased borderline and histrionic PD symptoms predicted decreased social skills [model fit: *χ*^2^ = 0.57, *df* = 28, *p* < 0.001, CFI = 0.979, TLI = 0.933, RMSEA = 0.042 (90% CI 0.030, 0.053)] (Table 3). Moreover, a higher level of parental antisocial PD at age 4 predicted declining social skills. Furthermore, no predictive effect of parental narcissistic PD symptoms was found with respect to either its intercept or slope. Although there were no observed associations between the level and change in overall (summed) symptoms of individual disorders belonging to Cluster A [model fit: *χ*^2^ = 66.72, *df* = 23, *p* < 0.001, CFI = 0.981, TLI = 0.956, RMSEA = 0.046 (90% CI 0.034, 0.058)] or C [model fit: *χ*^2^ = 66.00, *df* = 23, *p* < 0.001, CFI = 0.982, TLI = 0.948, RMSEA = 0.044 (90% CI 0.032, 0.057)] and change in social skills, the level of paranoid PD symptoms (Cluster A) and both a higher level and increase in avoidant PD symptoms (Cluster C) predicted reduced social skills.

**Table 3:** Change in social skills from age 4 to 10 years predicted from level (age 4) and change from age 4 to 6 in symptoms of each parental personality disorder

Predictor	*B* (95% CI)	*β*	*p*-value
Cluster A			
Paranoid—intercept	−0.27 (−0.53, −0.02)	−0.20	0.032
Paranoid—slope	0.12 (−0.36, 0.60)	0.04	0.63
Schizoid—intercept	0.03 (−0.22, 0.27)	0.01	0.84
Schizoid—slope	0.16 (−0.26, 0.57)	0.04	0.46
Schizotypal—intercept	0.02 (−0.18, 0.21)	0.01	0.88
Schizotypal—slope	−0.27 (−0.62, 0.09)	−0.11	0.14
Cluster B			
Antisocial—intercept	−0.26 (−0.51, −0.03)	−0.12	0.033
Antisocial—slope	−0.40 (−0.86, 0.04)	−0.09	0.083
Borderline—intercept	−0.08 (−0.22, 0.05)	−0.08	0.25
Borderline—slope	−0.39 (−0.650. −0.12)	−0.17	0.004
Histrionic—intercept	−0.12 (−0.35, 0.10)	−0.05	0.31
Histrionic—slope	−0.55 (−0.99, −0.11)	−0.13	0.018
Narcissistic—intercept	−0.02 (−0.19, 0.19)	−0.01	0.85
Narcissistic—slope	0.11 (−0.19, 0.53)	0.03	0.55
Cluster C			
Avoidant—intercept	−0.22 (−0.38, −0.06)	−0.17	0.006
Avoidant—slope	−0.46 (−0.79, −0.13)	−0.15	0.006
Dependent—intercept	−0.15 (−0.35, 0.06)	−0.09	0.16
Dependent—slope	−0.14 (−0.51, 0.24)	−0.04	0.48
OC—intercept	0.05 (−0.06, 0.16)	0.05	0.38
OC—slope	−0.06 (−0.29, 0.18)	−0.02	0.65

The intercept of social skills, parental anxiety and depression are included as covariates. Multivariable analyses in 3 models, one for each cluster, *OC* obsessive–compulsive personality disorder symptoms

## Discussion

Becoming socially competent is a prerequisite for favorable psychosocial development and adjustment in children, and improving social skills is a focus of a range of treatment and intervention efforts. Although there are several reasons to expect elevated PD symptoms in parents to make it more difficult for children to learn age-appropriate social skills, this possibility has never been examined. Drawing on a representative community sample followed up over 4 waves with children aged 4–10 years, we found that increased Cluster B PD symptoms, specifically borderline and histrionic PD symptoms, as well as avoidant PD (Cluster C) symptoms predicted decreased social skills in offspring, including when all unmeasured time-invariant confounding and time-varying levels of parental anxiety and depression were adjusted for. Additionally, more antisocial, avoidant, and paranoid PD symptoms predicted reduced social skills.

Previous studies have chronicled children of parents with Cluster B PDs to be at risk for a variety of problems, such as insecure attachment, emotion dysregulation, and poor mental health [6, 16, 41]; the majority of studies addressed borderline PD and, to some extent, antisocial traits in parents. We extend this research by showing that the acquisition of social skills in children may also suffer, that children of parents with histrionic symptoms are at risk as well, and that this potential intergenerational transmission is not attributable to time-invariant factors, such as shared genes or stable socio-economic factors and living conditions. Future work should detail the mechanisms by which parental PD symptoms affect the social skills of offspring, but we suggest three mechanisms. First, rapid shifts in emotions, unpredictability and self-preoccupation are expected in parents with symptoms of borderline, antisocial and histrionic PDs. Social skills enhancing parenting, such as sensitivity, consistency, scaffolding, and autonomy granting, may hence suffer [12, 15, 39]. Second, parents with these personality problems may also model ineffective ways of behaving in interpersonal relationships, including how to handle conflicts and intimacy, which their children adopt [38]. Third, such parental behavior may also compromise secure parent–child attachment [27, 29], thus leading children to develop internal working models that undermine socially competent behavior. Future studies should test these propositions and examine the full mediating chain from symptoms of PDs via parenting practices, modeling, and attachment to the social skills of offspring.

In addition, higher levels and increased parental avoidant PD symptoms predicted declines in children’s social skills. Avoidant PD is characterized by social inhibition and avoidance of interpersonal contact due to a fear of criticism and rejection and feelings of inadequacy [3]. Although avoidant PD has received far less attention than borderline and antisocial PDs, available evidence suggests that these avoidant PD features may be mirrored in the parent–child relationship [49], possibly through overprotection, intrusive control and messages to the child that the outside world is dangerous [48], thereby limiting children’s social interaction with peers and other adults from whom social skills can be learned and refined. Considering the substantial comorbidity between avoidant PD and social anxiety disorder, arguably reflecting that avoidant PD represents a severe version of social anxiety disorder [45], it is notable that symptoms of avoidant PD predicted reduced social skills even when the degree of parental anxiety was adjusted for. Possibly, the lack of intimacy, openness and self-respect typical of avoidant PD but not necessarily seen in social anxiety [14, 30] may limit children’s social experiences as well as model socially incompetent behavior.

Contrary to our hypothesis, increased symptoms of Cluster A, most Cluster C PDs and narcissistic PD (Cluster B) symptoms did not forecast decreased social skills. It is generally believed that the interpersonal problems inherent in PDs pervade all social relationships [3], but a recent meta-analysis concluded that in general, not all relationships are affected and that the ones that are afflicted vary between PDs [49]. Except for the present predictive effect of avoidant PD symptoms and the lack of risk found for children of parents with symptoms of paranoid PD, the parental PD symptoms that we found to entail risk for reduced offspring social skills overlap with the PDs that have been associated with impairment in the parent–child domain [49]. We extend these cross-sectional findings by demonstrating prospective impacts of parental symptoms of PDs, even when the model is adjusted for all time-invariant confounders and comorbid anxiety and depression. Nonetheless, even if we did not detect effects in late pre-school and the first years of school, the parenting that is necessary for acquiring social skills may be different from that which is necessary in middle childhood and adolescence. Sensitivity, responsiveness, and the provision of external emotion regulation may prove particularly important during early childhood [26]—capacities that are distinctly impaired in Cluster B disorders. In late middle childhood and adolescence, however, autonomy-granting and monitoring activities outside the family become increasingly relevant [15], and such parenting may be especially difficult for parents with Cluster A and C problems. Thus, the present findings, including the lack of effects of Cluster A and C PD symptoms, are confined to the age span studied and do not necessarily generalize to other developmental periods.

The finding that changes in parental antisocial and narcissistic PD symptoms do not predict child social skills is surprising given the severity of these disorders. However, a high level of antisocial PD symptoms for a parent with a child aged 4 did predict decreased social skills of the child. The intercept captures one’s level relative to that of others, and a range of unmeasured factors could influence it, including time-invariant factors, which might also impact future changes in social skills. Thus, causal interpretations are restricted compared to those possible with change–predicting–change results, as unmeasured time-invariant confounders are not ruled out. For example, shared genetic factors between parents and offspring could plausibly account for the effect.

### Strengths and limitations

This study has several strengths, such as the use of a large, representative community sample, longitudinal design, and a within-person approach to analyze longitudinal relations. However, some limitations should be noted. First, although there is little to indicate that PDs are categorical in nature [47], we cannot be certain that the present findings extend to the categorical conceptualization of PDs, because functional impairment was not considered. Nonetheless, it is noteworthy that even low or moderate PD symptoms can impact children. Second, information about PD symptoms was obtained through a self-report questionnaire, the DIP-Q. Although the DIP-Q ratings of symptoms concur with those obtained through diagnostic interviews [33], only a moderate level of correspondence has been reported for some disorders, particularly for narcissistic, schizoid and schizotypal symptoms. Hence, the present null findings with respect to these problems should be interpreted with caution. Third, parents reported on both their PD symptoms and their children’s social skills. Hence, correlations could emerge due to common methods. However, because we analyzed how changes in PD symptoms predicted changes in social skills, factors that did not change during the observation period (such as the rater) were adjusted for, thus limiting the impact of common methods. Fourth, although all unmeasured time-invariant factors and time-varying depression and anxiety were controlled, unmeasured time-varying factors may still influence and aggravate parents’ PD symptoms and children’s social skills. Individuals with PDs are often very sensitive to environmental changes, which can lead to profound emotional, behavioral, and cognitive reactions (American Psychiatric Association, 2013). Hence, time-varying factors, such as inter-parental conflicts and stressful life events, may not only be responsible for aggravating parents’ PD symptoms but also negatively impact children’s social skills [10, 25]. Finally, this study focused exclusively on the predictive power of PD symptoms for child social skills but did not assess the hypothesized mediating parenting factors, a task awaiting future research.

## Conclusion

The present study finds, for the first time, that an increase in parental symptoms of parental borderline, histrionic and avoidant PDs predicts a decrease in social skills in offspring from pre-school to middle childhood, including when all unmeasured time-invariant confounders and parental depression and anxiety are accounted for. Hence, service providers for both children and adults should be aware that such personality problems, even at subclinical levels, may interfere with children’s acquisition of age-appropriate social skills.
